# Introduction to “Advances in Photodynamic Therapy 2018”

**DOI:** 10.3390/molecules24010160

**Published:** 2019-01-03

**Authors:** Michael R Hamblin

**Affiliations:** 1 Wellman Center for Photomedicine, Massachusetts General Hospital, Boston, MA 02114, USA; hamblin@helix.mgh.harvard.edu; Tel.: +44-1835-824913; 2 Department of Dermatology, Harvard Medical School, Boston, MA 02115, USA; 3 Harvard-MIT Division of Health Sciences and Technology, Cambridge, MA 02139, USA

This Special Issue of *Molecules* entitled “Advances in Photodynamic Therapy 2018” contains seven papers, including five original reports and two reviews. As befits a chemistry-based journal, most of the papers cover photosensitizer structure and function in photodynamic therapy (PDT).

The first paper is a collaboration from the laboratories of Santi Nonell in Barcelona, Spain and Fatma Yurt in Izmir, Turkey [1]. These authors incorporated an imidazole-functionalized zinc-octa(*t*-butyl-phenoxy)-phthalocyanine inside mesoporous silica nanoparticles, and decorated them with Cetuximab, a monoclonal antibody that recognizes the epidermal growth factor receptor (EGFR). The goal was to target pancreatic cancer cells. Three different cell lines with different expression levels of EGFR were investigated. The antibody-conjugated nanoparticles (up to 5µM) in conjunction with red light fluences (up to 40 J/cm^2^) produced dose-dependent killing that correlated with EGFR expression levels. They hypothesized that the photosensitizer was transferred out of the nanoparticles into the cancer cells via protein binding.

The next paper [2] is from Ravi Pandey’s laboratory in the PDT Center, Roswell Park Cancer Institute, in Buffalo, NY. These authors report on PDT of mouse tumors (Colon26 in BALB/c mice) using four different photosensitzer (HPPH)-cyanine dye (Cypate or CD) conjugates. The idea was to use the photobleaching of the individual parts of the conjugate as surrogate markers for the generation of singlet oxygen during PDT, and the near-infrared (NIR) fluorescence for in vivo imaging and photobleaching. The best results in terms of percentage of tumors cured were obtained with a fluence of 128 J/cm^2^ delivered at a fluence rate of 14 mW/cm^2^, and these conditions correlated with the lowest photobleaching of the NIR dye.

The next paper [3] comes from the laboratories of Iou-Zen Chen and Ji-Yuan Liang in Taiwan. These authors studied a flavan-3-ol called catechin as a blue-light activated photosensitizer to kill Gram-negative bacteria *Acinetobacter baumannii* and a carbapenam-resistant variant. They found that superoxide radicals were produced under blue light illumination of catechin, which were responsible for the microbial killing, which could be inhibited by the addition of ascorbic acid.

A paper [4] from Jonathan Lovell’s laboratory at the State University of New York, Buffalo, NY, investigated whether a conjugate between meso-tetra(4-carboxyphenyl)porphyrin (mTCPP) and the amino acid, lysine, could be used to target cancer cells via the L-amino acid transporter. In order to prepare the tetravalent conjugates with either L-lysine or D-lysine, the Fmoc-protected amino-acids were used. Their in vitro results with U87 cells showed that neither the L-lysine nor the D-lysine derivatives of mTCPP were taken up by the cells or mediated PDT killing. However, both the Fmoc-protected derivatives displayed non-specific cellular uptake and good PDT killing.

The next paper [5] is a collaboration between laboratories from Jinan, China and Brasilia, Brazil. It reports the synthesis of five novel benzo[a]phenoxazinium chlorides as photosensitizers against 4T1 mouse breast cancer cells and NIH 3T3 mouse fibroblasts. One of the compounds was the first reported benzo[a]phenoxazinium dimer. The compounds showed a range of toxicity and phototoxicity towards cancer and normal cells, and the dimeric compound showed the most selective PDT killing of the cancer cells while sparing normal cells.

There follows a review paper [6] by the guest editor of the Special Issue, Michael R Hamblin, together with Heidi Abrahamse from Johannesburg in South Africa. It describes a series of studies from the Hamblin laboratory at the Wellman Center for Photomedicine at MGH in Boston, MA. These studies concern antimicrobial photodynamic inactivation using a range of different photosensitizers, and the potentiation of bacterial killing by a variety of inorganic salts. The paper concentrates on the different photochemical mechanisms that operate. For instance, sodium azide works by type I electron transfer to form azide radicals, while potassium iodide works via type II singlet oxygen reaction with iodide to form free iodine and iodine radicals. Potassium bromide only works with titanium dioxide photocatalysis. Sodium thiocyanate works via a combination of type II and type I pathways to form sulfur dioxide radical anions, while sodium selenocyanate works via the type II pathway to form selenocyanogen. Sodium nitrite appears to work via a type II mechanism to form peroxynitrate.

The last paper [7] is a comprehensive review paper with 229 references from the laboratory of Celine Frochot at the University of Lorraine in Nancy, France. The subject is the use of cyclodextrins (CD) in PDT. CD are naturally occurring cyclic oligosaccharides produced during the bacterial digestion of cellulose, which consist of between six and eight (α-1,4)-linked α-d-glucopyranose units. These vehicles are particularly effective at dissolving and disaggregating hydrophobic photosensitizers (PS) within the hydrophobic pocket of the CD. The review is divided into three parts covering the following: (1) non-covalent CD–PS inclusion complexes, covalent CD–PS conjugates, and CD–PS nanoassemblies; (2) incorporating CD–PS systems into hybrid nanoparticles (NP) using upconverting or other types of NP; and (3) CDs with fullerenes as PS. 
